# Optimal Cardiac Resynchronization Therapy with Conduction System Pacing Guided by Electro-Anatomical Mapping: A Case Report

**DOI:** 10.3390/jcdd10110456

**Published:** 2023-11-09

**Authors:** Catalin Pestrea, Roxana Enache, Ecaterina Cicala, Radu Vatasescu

**Affiliations:** 1 Interventional Cardiology Unit, Brasov County Clinical Emergency Hospital, 500326 Brasov, Romania; spac.roxana@yahoo.com (R.E.); cicalaecaterina@gmail.com (E.C.); 2 Department of Medical and Surgical Specialties, Faculty of Medicine, “Transilvania” University of Brasov, 500019 Brasov, Romania; 3 Faculty of Medicine, Carol Davila University of Medicine and Pharmacy, 050474 Bucharest, Romania; radu.vatasescu@umfcd.ro; 4 Electrophysiology and Cardiac Pacing Lab, Clinical Emergency Hospital, 014461 Bucharest, Romania

**Keywords:** cardiac resynchronization therapy, His bundle pacing, left bundle branch area pacing, 3D mapping

## Abstract

Introduction: Biventricular pacing has been the gold standard for cardiac resynchronization therapy in patients with left bundle branch block and severely reduced left ventricular ejection fraction for decades. However, in the past few years, this role has been challenged by the promising results of conduction system pacing in these patients, which has proven non-inferior and, at times, superior to biventricular pacing regarding left ventricular function outcomes. One of the most important limitations of both procedures is the long fluoroscopy times. Case description: We present the case of a 60-year-old patient with non-ischemic dilated cardiomyopathy and left bundle branch block in whom conduction system pacing was chosen as the first option for resynchronization therapy. A 3D electro-anatomical mapping system was used to guide the lead to the His bundle region, where correction was observed at high amplitudes, and afterward to the optimal septal penetration site. After reaching the left endocardium, left bundle branch pacing achieved a narrow, paced QRS complex with low fluoroscopy exposure. The three-month follow-up showed a significant improvement in clinical status and left ventricular function. Conclusion: Since conduction system pacing requires a great deal of precision, targeting specific, narrow structures inside the heart, 3D mapping is a valuable tool that increases the chances of success, especially in patients with complex anatomies, such as those with indications for cardiac resynchronization therapy.

## 1. Introduction

Biventricular pacing (BVP) has been the gold standard for cardiac resynchronization therapy (CRT) in patients with left bundle branch block (LBBB) and severely reduced left ventricular ejection fraction (LVEF) for decades [1]. However, in the past few years, this role has been challenged by the promising results of conduction system pacing (CSP) in these patients. CSP proved consistently that the generated paced QRS complex was significantly shorter than the one produced by BVP. This observation was probably responsible for the non-inferiority and, at times, superiority compared to BVP regarding left ventricular function outcomes [2,3]. Both procedures have certain limitations. While BVP success is limited by a significant number of non-responders (up to 30%) and is highly dependent on coronary sinus anatomy, CSP is less effective in very distal conduction disorders and is technically challenging in diseased hearts. In addition, both procedures frequently require long fluoroscopy times for lead positioning [4]. We present a case of CRT in which 3D electro-anatomical mapping guided initial His bundle pacing (HBP) followed by left bundle branch area pacing (LBBAP) with an optimal electrical and mechanical response. 

## 2. Case Description

**Figure 1:**
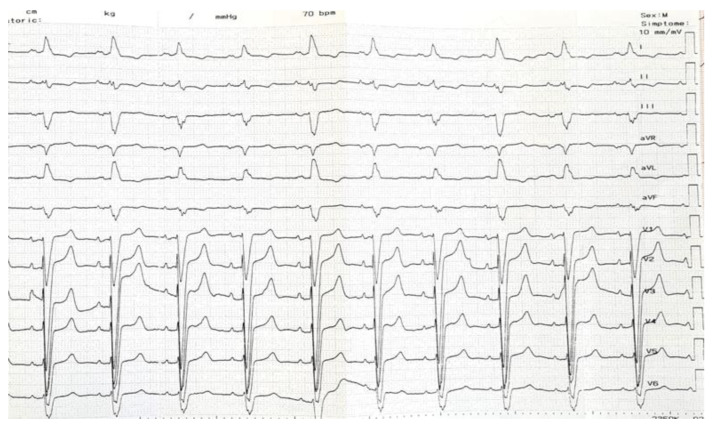
Initial ECG recording showing sinus rhythm with a left bundle branch block morphology. ECG, electrocardiogram. ECG–electrocardiogram.

**Figure 2:**
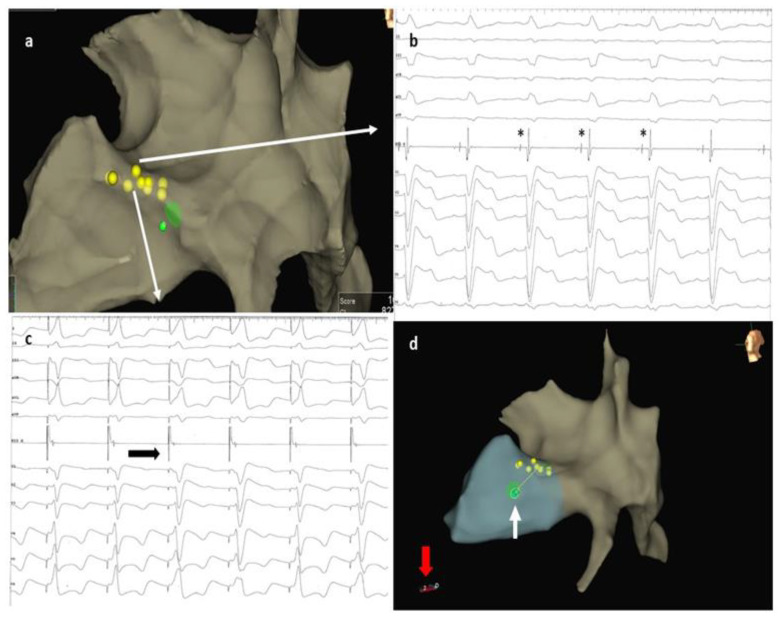
(**a**) 3D mapping image showing reconstruction of the right atrium, the basal right ventricle, and the His cloud (yellow dots). The tip lead is visible as a green dot. (**b**) Basal 12 lead ECG showing a left bundle branch block morphology and electrogram recorded at the distal part of the His (white arrow) with a prominent HB potential marked with *. (**c**) Pacing at that site revealed a transition from non-selective HBP with LBBB correction to myocardial pacing (black arrow) at high amplitudes (see text for explanations). (**d**) After HBP failure, the ideal site for septal penetration situated 1.5 cm towards the apex was marked on the 3D system (blue dot). Note the atrial lead placed at the RV apex (red arrow). ECG, electrocardiogram; HB, His bundle; HBP, His bundle pacing; LBBB, left bundle branch block; RV, right ventricle.

**Figure 3:**
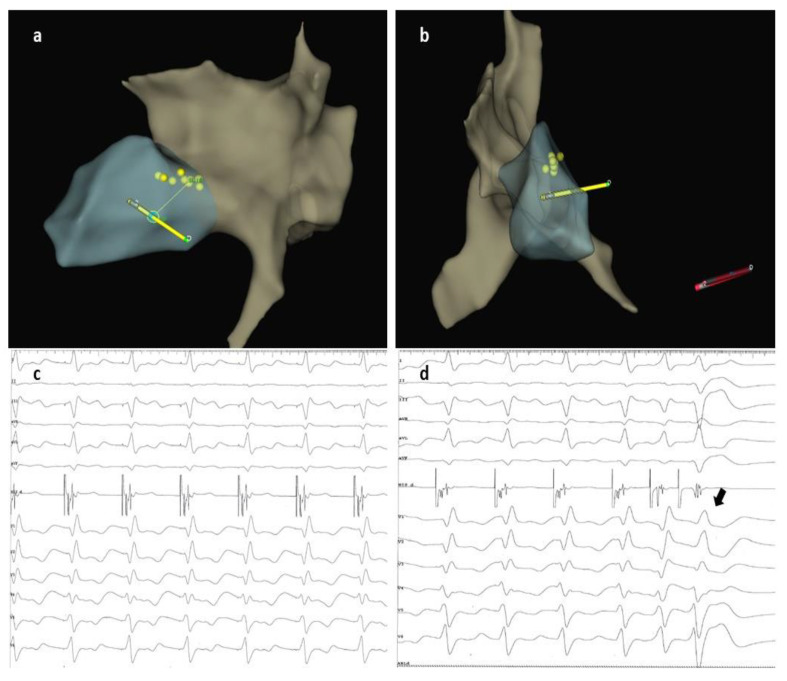
(**a**,**b**) 3D images showing the advancement of the lead into the septum through the previously marked blue dot. (**c**) Pacing from the lead tip revealed a narrow QRS complex with a QR morphology in lead V1, a duration of 125 ms, and an LVAT of 70 ms. (**d**) Differential pacing with two extra stimuli showed an evident change in morphology (black arrow), thus proving LBB capture. LVAT, left ventricular activation time; LBB, left bundle branch.

**Figure 4:**
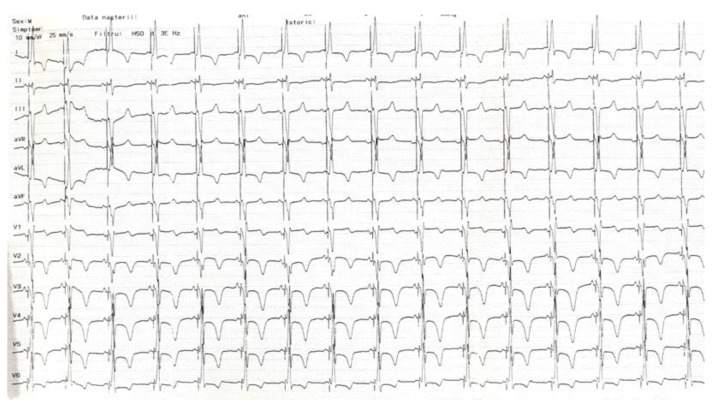
Final ECG showing atrial synchronized narrow-paced QRS complexes with different degrees of fusion (lead V1). ECG, electrocardiogram.

A 60-year-old male patient diagnosed with non-ischemic dilated cardiomyopathy with reduced LVEF and LBBB was referred to our clinic for progressive exertional dyspnea and fatigue symptoms developed over the previous two years. The patient was in functional NYHA class II despite maximally tolerated doses of angiotensin receptor-neprilysin inhibitors, beta-blockers, mineralocorticoid receptor antagonists, and sodium-glucose cotransporter-2 inhibitors for the past six months. The presenting ECG showed sinus rhythm with an LBBB morphology and a QRS duration of 160 ms (Figure 1). Echocardiography revealed an enlarged left ventricle (LV) with an ejection fraction of 26%, normal wall thickness, overt signs of intraventricular dyssynchrony (apical rocking and septal flash), moderate mitral regurgitation, biatrial enlargement, and non-dilated right ventricle. The lab results were unremarkable. Given the clinical, electrocardiographic, and echocardiographic findings, the patient had a class I recommendation for CRT. Our laboratory has adopted CSP as the first option for patients with CRT indications for the past two years. For this case with potentially difficult anatomy (dilated LV and atria possibly displacing and modifying the trajectory of the conduction system), we decided to perform the procedure guided by a 3D mapping system, the Ensite Precision system (Abbott Cardiovascular, Plymouth, MN, USA), to reduce the X-ray exposure. After obtaining two separate entry sites in the axillary vein, a conventional atrial lead was placed at the right ventricular apex and connected to the 3D system to serve as a reference and backup pacing. Using the other axillary route, a deflectable decapolar EP catheter was used to create the anatomy of the right atrium, the coronary sinus, and the basal part of the right ventricle. After delineating the tricuspid valve, we thoroughly mapped and tagged the His bundle (HB) cloud from proximal to distal (Figure 2a). With the map completed, the EP catheter was withdrawn, and a deflectable Medtronic C304 His catheter (Medtronic Inc., Minneapolis, MN, USA) with a Medtronic SelectSecure 3830 lead (Medtronic Inc., Minneapolis, MN, USA) inside was introduced. The lead was connected in a unipolar fashion to the 3D system so that the tip of the lead would be visible on the map (Figure 2a). The catheter was placed at the distal part of the HB cloud (Figure 2b), where repeated pacing showed complete correction of the LBBB, but unfortunately, at unacceptably high thresholds (3 V at 1ms pulse duration) (Figure 2c). In the next step, a point was marked on the 3D map at 1.5 cm from the distal His location towards the right ventricular apex (Figure 2d). The catheter was placed at that spot, and the lead was screwed deep into the septum under minimal fluoroscopic guidance until fixation beats with right bundle branch block morphology were observed (Figure 3a,b). Pacing at that site revealed a narrow QRS complex with a QR morphology in lead V1, a duration of 125 ms, and an LVAT of 70 ms (Figure 3c). Differential pacing with two extra stimuli showed an evident change in the morphology of the premature complex, proving the initial capture of more than one structure and, implicitly, the LBB capture (Figure 3d). The catheter was retracted and slit, followed by atrial lead placement in the right atrial appendage under fluoroscopy. The procedural pacing threshold was 0.75 V at 0.4 ms pulse duration with a detection of 12 mV and a fluoroscopy time of 2 min. The total procedural time was 120 min, with 17 min dedicated to 3D mapping. The final electrocardiography showed atrial synchronized, narrow-paced QRS complexes with different degrees of fusion (Figure 4). There were no periprocedural complications, and the patient was discharged uneventfully the next day. The pacing and sensing thresholds were stable over the follow-up period. The 3-month echocardiography follow-up revealed a significant increase in LVEF (from 26% to 43%) and a decrease in left ventricular volumes (the end-systolic volume decreased from 174 mL to 132 mL). The patient also had a substantial clinical improvement with no heart failure symptoms during normal daily activities.

## 3. Discussion

In both BVP and CSP, the final target position for lead placement is commonly assessed under fluoroscopy. In BVP, fluoroscopy is used for coronary sinus angiography, which is crucial to identifying the destination target vein (ideally in the postero-lateral part of the LV), followed by deploying an over-the-wire lead in that territory. On the other hand, the trajectory of the conduction system in physiological pacing is assessed using indirect fluoroscopic markers, like the tricuspid valve, the HB location, and the right ventricular apex, thus being subjected to less precision in some patients.

Besides the harmful effect of X-ray exposure on doctors and patients, there are several limits to two-dimensional fluoroscopy-only guided CSP procedures for CRT. First, most patients with heart failure and LBBB have a modified anatomy, enlarged atria and/or ventricles, and valvular regurgitations, resulting in an anteriorly and potentially distal displacement of the HB position. We have shown in a previous study that enlarged atria are significantly associated with failure to identify the HB electrogram [5]. On the other hand, since existing data states that in up to 60% of the LBBB patients, the site of the block is within the HB itself or the proximal LBB, we believe it is worthwhile to search initially for the HB [6]. Mapping the HB with a deflectable EP catheter has some advantages. Without worrying about X-ray exposure, after recreating the anatomy of the right atrium and the basal right ventricle, a complete His cloud can be recorded from the proximal to the very distal part, highlighting the points where pacing achieved complete correction, thus serving as an ideal target for the pacing lead. Furthermore, by analyzing the 3D map of the right atrium and the position of the HB and the tricuspid valve, a proper selection of delivery catheters can be made, opting for either non-deflectable catheters with a specific proximal curve or a deflectable catheter for more challenging anatomies.

In our case, LBBB correction was observed during distal HBP at high amplitudes, proving the proximal localization of the site of the block. Although the site was not accepted due to concerns over fast battery depletion and the risk of further pacing threshold increase over time, this finding showed us that CSP would be efficient in this patient, with the important condition of capturing the dormant LBB fibers while pacing the left endocardium.

In this regard, 3D mapping provided the opportunity to mark the best site on the right side of the interventricular septum to initiate perforation, which is, based on the anatomical course of the LBB, between 1 to 2 cm from the HB position towards the ventricular apex. We marked the position of the apex by temporarily placing the atrial lead there, thus avoiding extensive and useless right ventricular mapping. Conventionally, the optimal site for penetrating the septum was chosen fluoroscopically and by paced QRS morphologies [7]. However, this may lead to more superior or inferior positions, missing the bulk of the left bundle ramifications [8]. Since only the tip of the lead was visible on the 3D system (the proximal electrode is inside the sheath), and to ensure proper contact and perpendicularity of the catheter on the septum, we advanced the lead into the septum with a minimum amount of fluoroscopy. Afterward, with the catheter retracted, the distal part of the lead is visible, and the depth of penetration and the perpendicularity of the lead on the septum can be easily assessed.

Previous case reports and an observational study using the same 3D mapping system have been published on electro-anatomically guided CSP with overall good results, but still with long fluoroscopy times, contradicting the original idea of a low fluoroscopy procedure [9,10]. In another prospective study that included 32 patients with advanced conduction abnormalities and structural heart disease, electro-anatomically guided LBBAP was achieved in 91% of the patients with a mean fluoroscopy time of 0.93 min. Of note, in that study, the physicians did not use an electrophysiology catheter to map the heart, instead using only the lead to identify the HB location [11]. An obvious advantage to creating a voltage map of the septum would be identifying a scarred area where the lead would be less likely to penetrate. On the other hand, a completely zero-fluoroscopy procedure is difficult to achieve since vascular access, atrial lead placement, sheath retraction, and slitting may require fluoroscopy. In our case, the entire procedure was performed within 2 min of fluoroscopy while acknowledging that septal perforation was performed under fluoroscopic guidance and achieved on the first attempt. A fluoroless approach to septal perforation is possible, but we wanted to increase the chance of success from the first attempt. Most certainly, initial failure with several subsequent attempts would have increased the total fluoroscopy time.

One limitation of this case report is that although our center is very experienced with fluoroscopy-guided CSP, we recently implemented 3D mapping guidance, so our experience is limited to a few initial cases. Therefore, with increasing expertise in this technique, we expect further improvements in procedural outcomes, including reductions in fluoroscopy time. The total procedural time is influenced by the time spent recreating the cardiac 3D map. In our case, we opted for detailed mapping of the right atrium, the conduction system, and the basal right ventricle. This increased the total duration of the procedure by nearly 15 min. If one maps only the critical elements for CSP, like the HB, the procedure would be significantly shorter. Knowing the precise location of the conduction system may reduce the time spent otherwise using fluoroscopy to place the lead at the desired site, so the total time with 3D mapping and fluoroscopy guidance may not be that different. Overall, in our experience, the total procedural duration was not significantly longer than a conventional LBBAP procedure.

The take-home message from this case report would be that CSP is, in the end, a precision procedure, and every technical feature that highlights the localization of the discrete target area increases the success odds. Electro-anatomical mapping, on top of the electrophysiological findings, provides in a safe environment the necessary landmarks to navigate different parts of the conduction system, especially in patients with baseline diseased hearts and modified anatomies.

Using any means to increase the success rate for CSP in patients with CRT indications may provide benefits beyond improving the LV function. Although no comparative studies have been performed on this matter, CSP may have a more positive impact on the psychological profile of the patients since factors like phrenic nerve stimulation, which are frequently encountered with BVP and affect the quality of life, are absent [12]. In addition, it has been shown that BVP improves cognitive performance in patients with heart failure [13]. CSP, using the same arguments of an increased LV function and better cerebral perfusion, is expected to be associated with the same outcomes, but future studies on this topic are required.

## 4. Conclusions

Since CSP requires a great deal of precision, targeting specific, narrow structures inside the heart, 3D mapping is a valuable tool that increases the chances for success, especially in patients with complex anatomies, such as those with indications for CRT. The major advantage of this technique is a significant reduction in fluoroscopy time, and with increasing expertise in this technique, further improvements in procedural outcomes could be expected.

## Data Availability

The data are available from the authors upon reasonable request.
